# Network meta-analysis of pharmacological treatment for antibody-mediated rejection after organ transplantation

**DOI:** 10.3389/fimmu.2024.1451907

**Published:** 2024-12-12

**Authors:** Junjie Sun, Yanqing Yu, Fu Huang, Qiuwen Zhang, Lirong Zhu, Guining He, Haibin Li, Xuyong Sun

**Affiliations:** ^1^ Institute of Transplantation Medicine, The Second Affiliated Hospital of Guangxi Medical University, Guangxi Clinical Research Center for Organ Transplantation, Guangxi Key Laboratory of Organ Donation and Transplantation, Nanning, Guangxi, China; ^2^ Department of Urology, The Second Affiliated Hospital of Guangxi Medical University, Nanning, China

**Keywords:** antibody-mediated rejection, organ transplantation, pharmacological treatments, network meta-analysis, randomized controlled trials

## Abstract

**Objective:**

This study aims to assess the efficacy of pharmacological interventions in mitigating graft injury in transplant patients with antibody-mediated rejection (AMR) through a network meta-analysis (NMA).

**Methods:**

A search was conducted on databases such as Cochrane Library, PubMed, EmBase, and Web of Science for randomized controlled trials (RCTs) on pharmacological interventions for alleviating graft injury following AMR. The search was performed for publications up to April 12, 2024. Two reviewers conducted independent reviews of the literature, extracted data, and assessed the risk of bias (ROB) in the included studies using the ROB assessment tool recommended by the Cochrane Handbook for Systematic Reviews of Interventions 5.1.0. A Bayesian NMA was conducted using R 4.4.0, RStudio software, and the GeMTC package to assess the outcomes in estimated glomerular filtration rate (eGFR), mean fluorescence intensity (MFI), g-score, and infection under pharmacological treatments.

**Results:**

A total of 8 RCTs involving 215 patients and 6 different pharmacological treatments were included in this NMA. The results indicated that the increase in eGFR by eculizumab (SUCRA score: 81) appeared to be more promising. The decrease in MFI by bortezomib (SUCRA score: 72.3), rituximab (SUCRA score: 68.2), and clazakizumab (SUCRA score: 67.1) demonstrated better efficacy. The decrease in g-score by eculizumab (SUCRA score: 74.3), clazakizumab (SUCRA score: 72.2), and C1INH (SUCRA score: 63.6) appeared to have more likelihood. For infection reduction, clazakizumab (SUCRA score: 83.5) and bortezomib (SUCRA score: 66.8) might be better choices.

**Conclusion:**

The results of this study indicate that eculizumab has the potential to enhance eGFR and reduce g-score. Bortezomib demonstrates superior efficacy in reducing MFI. Clazakizumab appears to be more effective in reducing infections.

**Systematic review registration:**

https://www.crd.york.ac.uk/prospero/, identifier CRD42024546483.

## 1 Introduction

For end-stage diseases, organ transplantation has emerged as the therapy of choice (1, 2). However, antibody-mediated rejection (AMR) is the main cause of late allograft loss in solid organ transplantation (3, 4). Transplantation physicians have shifted their attention towards AMR and late allograft alterations. Although the clinical understanding of acute and chronic AMR has grown, the intricacies of diagnosis and histopathology, the absence of established treatment protocols, and the uncertainty surrounding long-term consequences make it challenging to identify and manage AMR in a timely manner (5). Currently, the standard of care (SOC) for AMR includes plasmapheresis and intravenous immunoglobulin (IVIG). Additionally, transplant recipients have also achieved successful treatment of AMR using agents that target B cells (rituximab), plasma cells (bortezomib), and the complement system (eculizumab) (6). To date, no specific therapy can address all facets of the alloimmune response (7). Once the late AMR occurs, it is often irreversible (8). Consequently, the incidence of graft damage or even failure caused by AMR has not declined significantly among transplant patients (9). Statistically, about 40% of transplants fail within ten years of transplantation (10, 11). This must be taken into account for retransplantation (8). However, the success rate of retransplantations is often lower than that of primary transplants (12, 13). This will have a significant impact on individuals, families, and society (14). It is therefore essential to develop safe and effective treatment options to protect graft functions in transplant patients with AMR. In recent years, there has been a growing body of studies focusing on pharmacological treatments to mitigate graft impairment after AMR. However, the comparative advantages of these treatments remain uncertain (6, 15).

Network meta-analysis (NMA) enables indirect comparisons and quantitative evaluation of different treatments to determine the most effective approach (16). This study employs NMA to investigate the impact of pharmacological treatments on alleviating graft injury in transplant patients with AMR. The goal is to provide guidance for clinical practice.

## 2 Data and methods

This study has been registered on the PROSPERO platform with the registration number CRD42024546483. Details are provided in the PRISMA extension statement for reporting of systematic reviews incorporating NMAs of health care interventions: checklist and explanations (16).

### 2.1 Literature screening

A combination of Medical Subject Headings (MeSH) terms and free-text terms (including AMR, humoral rejection, and RCTs) were applied to retrieve studies from databases such as Cochrane Library, PubMed, EmBase, and Web of Science up to April 12, 2024. The specific search strategy is shown in **Supplementary Material 1**.

### 2.2 Inclusion and exclusion criteria

#### 2.2.1 Study type: RCTs

##### 2.2.1.1 Study population: organ transplantation patients with AMR.

###### 2.2.1.1.1 Intervention measures

Test group: Bortezomib therapy, clazakizumab therapy, C1INH therapy, eculizumab therapy, rituximab therapy, and rituximab-Ig therapy. Control group: SOC or placebo. The control group received SOC treatments such as plasmapheresis/IVIG or placebo.

#### 2.2.2 Outcome measures

*Primary outcomes* (1): Estimated glomerular filtration rate (eGFR). (2) Mean fluorescence intensity (MFI). (3) G-score (17, 18).

Secondary outcome: Infection.

#### 2.2.3 Exclusion criteria

(1) Non-English literature. (2) Duplicate publications. (3) Studies with a lack of usable outcome measures. (4) Data errors or unobtainable data, even after attempting to contact the authors. (5) Test or control groups receiving drugs for inductive treatment.

### 2.3 Data extraction

Two reviewers were responsible for screening studies, extracting data, and cross-verifying the information independently. In the event of any discrepancies, it was resolved through discussion or consultation with a third reviewer. The studies were initially screened by reading the title. After excluding obviously irrelevant studies, the abstracts and full texts were further examined to determine which studies were eligible. If necessary, attempts were made to contact the original authors of these studies via email or phone to obtain critical information that was uncertain but vital for this study. The extracted data included: (1) Basic information of included studies: study title, first author, country, publication year, etc. (2) Baseline characteristics of study subjects and interventions. (3) Outcome measures of interest and outcome measurements.

### 2.4 Quality assessment

Two reviewers independently assessed the risk of bias (ROB) of included RCTs using the ROB assessment tool recommended by the Cochrane Handbook version 5.1.0. The results were cross-verified, and any discrepancies were resolved through discussion or consultation with a third reviewer. The assessment covered seven key areas (adequate sequence generation; allocation concealment; blind approach of outcome evaluators blinding; incomplete outcome data and how it was addressed; selective reporting of the outcome; and any other biases). Each item was rated as low risk, high risk, or unclear risk.

### 2.5 Statistical analysis

This study employed a Bayesian NMA that was conducted using R version 4.4.0, RStudio software, and the GeMTC package. After the consistent and non-consistent modeling, the Deviance Information Criterion (DIC) is detailed in **Supplementary Material 2**. The results demonstrated the consistency of the model’s performance. Fixed-effects models were employed to summarize the effect estimates from different studies. The combined effect size was described using the mean difference (MD) and a 95% confidence interval (CI). The results were presented in the form of forest plots, league tables, and cumulative probability ranking plots. The Surface Under the Cumulative Ranking Curve (SUCRA) was calculated to indicate the likelihood of each intervention being the most effective. SUCRA values were calculated on a scale of 0 to 1, with interventions ranked in order of their SUCRA values. Network diagrams and comparison-adjusted funnel plots were created using Stata 18. The publication bias (PB) risk was presented using Stata 18.

## 3 Results

### 3.1 Process and results of literature search

**Figure 1:**
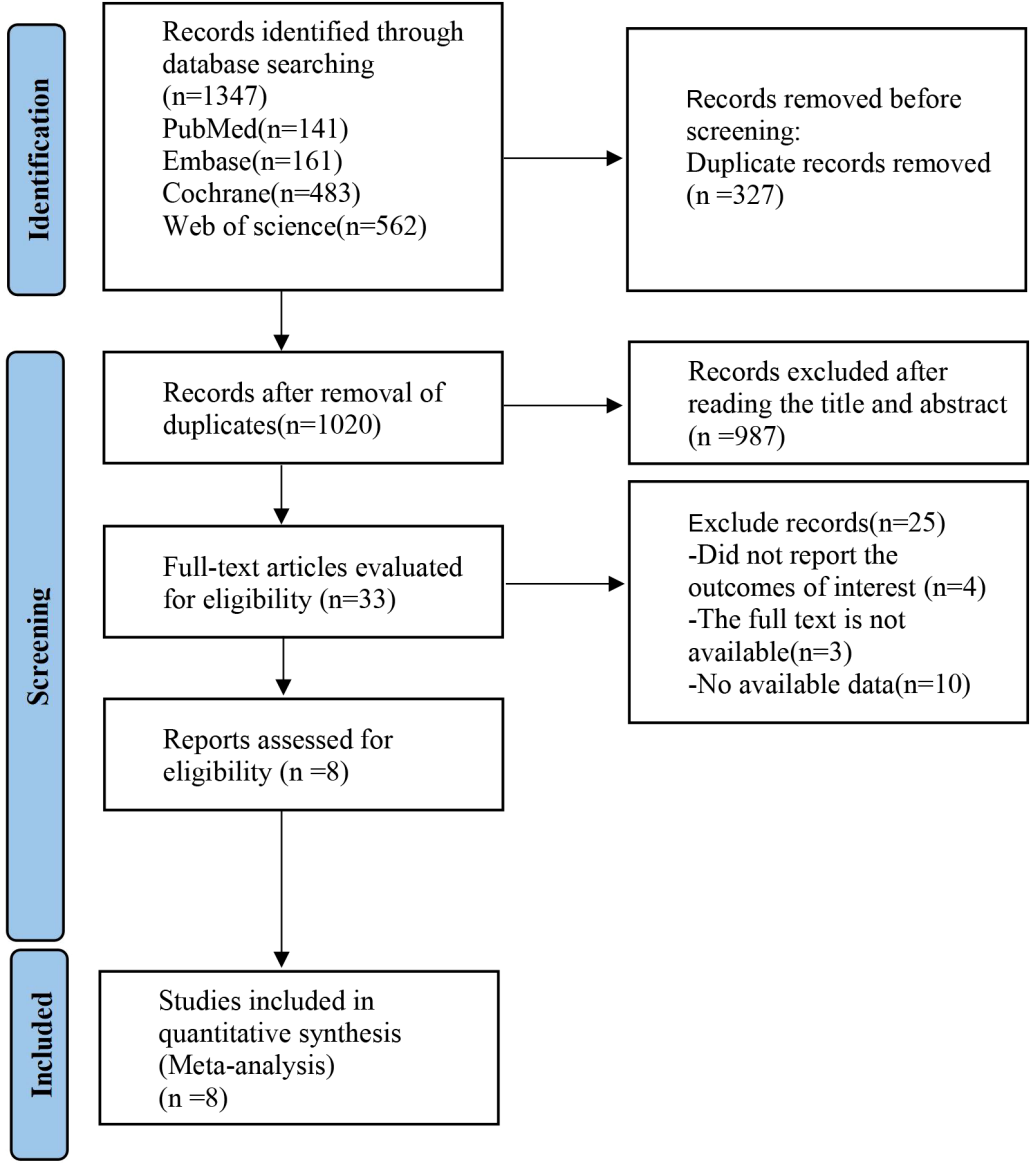
PRISMA flow diagram of the study process. PRISMA, Preferred Reporting Items for Meta-analysis.

The initial screening yielded a total of 1,347 relevant English articles. After a multi-step selection process, 327 duplicates were removed, 987 articles were excluded based on title and abstract review, 25 articles were excluded after reading the full text, and 8 articles were included in the final analysis. The screening process and results are detailed in **Figure 1**.

### 3.2 Baseline characteristics of included studies

Eight (8) RCTs were ultimately included, with a total of 215 patients. Among these, two RCTs were from America, two from France, two from Austria, and one from South Korea and Spain each. Six pharmacological interventions were utilized, i.e., bortezomib (2), clazakizumab (1), C1INH (9), eculizumab (4, 5), rituximab (3, 6), and rituximab-Ig (8). All of the interventions were compared in pairs with the control group (SOC or placebo). The basic characteristics of included studies are detailed in **Table 1**.

**Table 1:** The basic characteristics of included studies.

Study	Year	Country	Sample size	Gender (M/F)	Organ	Mean age	Intervention	Outcome
1.Konstantin Doberer	2021	Austria	Clazakizumab: 10 Control (placebo): 10	10/10	Kidney	Clazakizumab: 37.4, IQR (27.1–57.9) Control:31.4 (22.3–42.3)	Clazakizumab: 25 mg IV (4-weekly)	F1 F6 F7 F9
2.Farsad Eskandary	2018	Austria	Bortezomib: 21 Control (placebo): 23	18/26	Kidney	Bortezomib: 49.1, IQR (28.6–55.2) Control:45.5 (35.2–54.0)	Bortezomib: 1.3 mg/m2 IV on days 1, 4, 8, and 11)	F1 F6 F7 F9
3.Elodie Bailly	2020	France	Rituximab: 27 Control (placebo): 11	21/17	Kidney	Rituximab: 48 ± 16 Control: 40 ± 15	Rituximab: 375 mg/m2 IV	F1 F6 F7
4.S. Kulkarni	2017	America	Eculizumab: 10 Control (placebo): 5	9/6	Kidney	Eculizumab: Median (range) 44 (33–65) Control: 38 (20–57)	Eculizumab: 900 mg IV	F1 F6
5.Sujung Heo	2022	South Korea	Eculizumab: 7 SOC (plasmapheresis/intravenous immunoglobulin)): 4	3/8	Kidney	Eculizumab: 43.1 SOC: 45.7	Eculizumab: 900 mg IV	F1 F7
6.Bénédicte Sautenet	2016	France	Rituximab: 19 Control (placebo): 19	21/17	Kidney	Rituximab: 44.6 ± 16.8 Control: 46.7 ± 16.2	Rituximab: 375 mg/m2 IV	F1 F9
7.R.A. Montgomery	2016	America	C1INH: 9 Control (placebo): 9	7/11	Kidney	C1 INH: 48.6 ± 12.5 Control (placebo): 48.8 ± 13.0	C1INH: 5000 U IV	F1 F9
8.Francesc Moreso	2018	Spain	Rituximab-IG: 12 Control (placebo): 13	15/10	Kidney	Rituximab-IG: 47 ± 13 Control (placebo): 49 ± 15	Rituximab-IG: IG 0.5 g/kg IV and Rituximab 375 mg/m2 IV	F1 F6 F7 F9

M/F, Male/Female; Rituximab-IG, Intravenous rituximab and immunoglobulins; F1, Infections; F6, Estimated glomerular filtration rate(eGFR); F7, Mean fluorescence intensity (MFI); F9, G-score.

### 3.3 Network relationships of interventions

**Figure 2:**
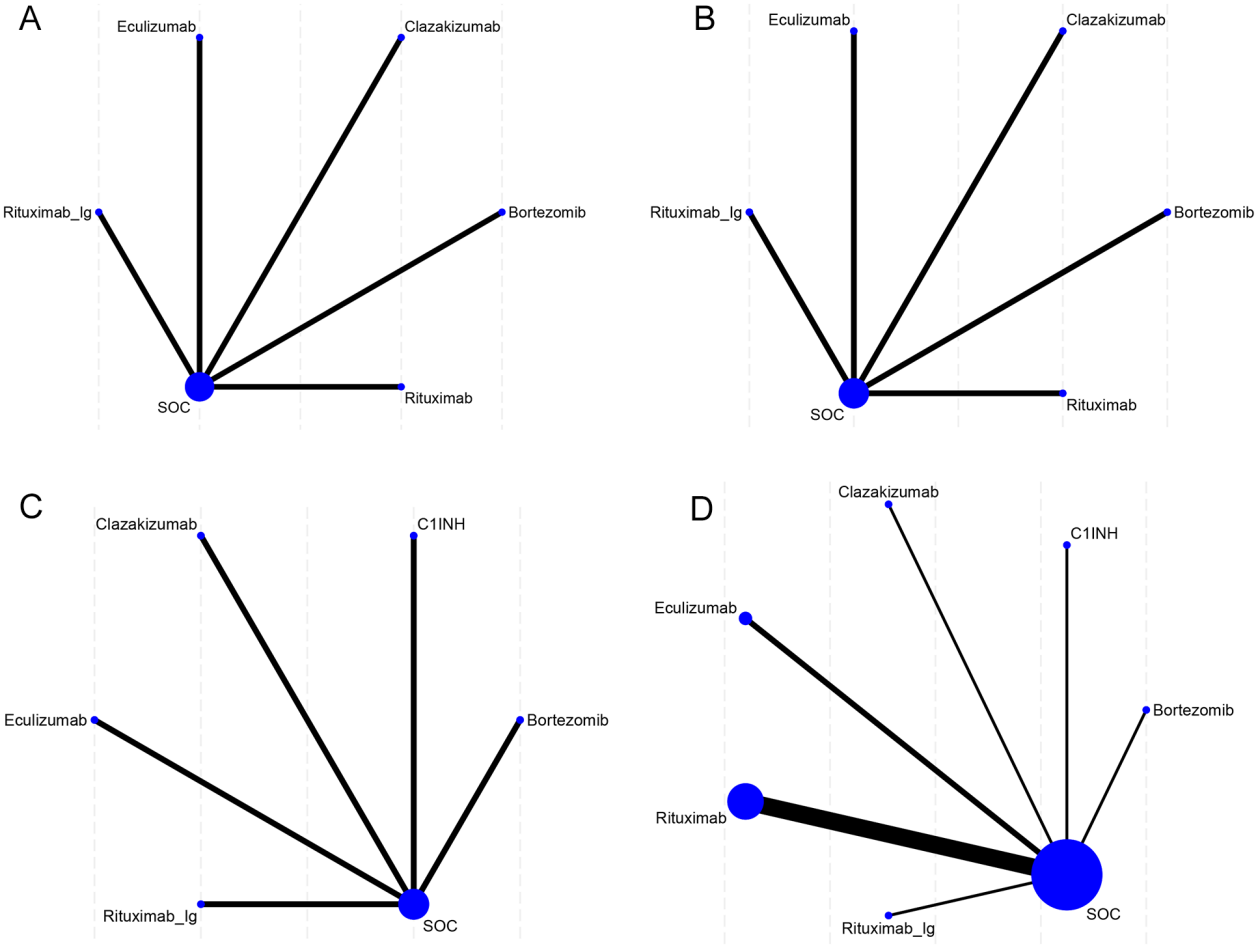
Network meta-analysis maps of the studies accessing the efficacy of AMR in the context of drug therapy on **(A)** EGFR, **(B)** MFI, **(C)** G-score, **(D)** Infection. The size of the nodes relates to the number of participants in that intervention type, and the thickness of lines between the interventions relates to the number of studies for that comparison.

The network diagram (**Figure 2**) of interventions displays all comparisons available for the included studies. A direct relationship is indicated by a line between two circles, while no line indicates no direct relationship. The size of the circles represents the sample size of the interventions, and the thickness of the lines represents the number of studies included between the two interventions. In this study, 1 (5) RCT compared eculizumab with SOC, and 7 (1-4, 6-8) RCTs compared clazakizumab, bortezomib, rituximab, eculizumab, rituximab, C1INH, and rituximab-Ig with placebo, respectively.

### 3.4 ROB assessment diagram

**Figure 3:**
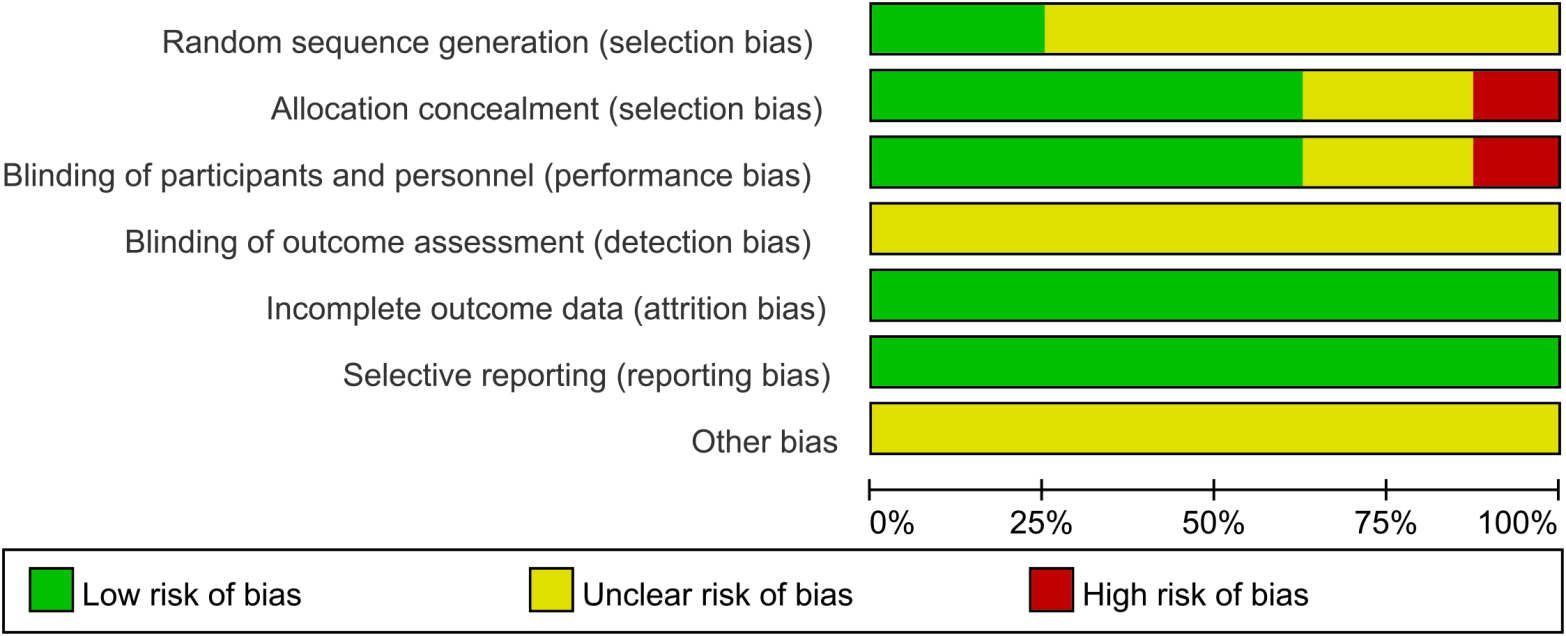
Bias risk assessment chart.

A total of 8 studies were included in this NMA. Among the studies, 2 employed randomization techniques, such as random number tables or envelope methods, while the remaining 6 studies simply mentioned the term “random”. Five studies provided comprehensive descriptions of the methods employed for concealed sequence allocation. Five studies reported the implementation of double-blinding. Not a single study mentioned the blinding of outcome assessors. Five studies provided details regarding subject dropout, including the groups with subject dropout and the specific reasons for withdrawal. All included studies exhibited a relatively low probability of selective reporting bias and other sources of bias. The ROB assessment results of included studies are presented in **Figure 3**.

### 3.5 Results of NMA

#### 3.5.1 EGFR

**Figure 4:**
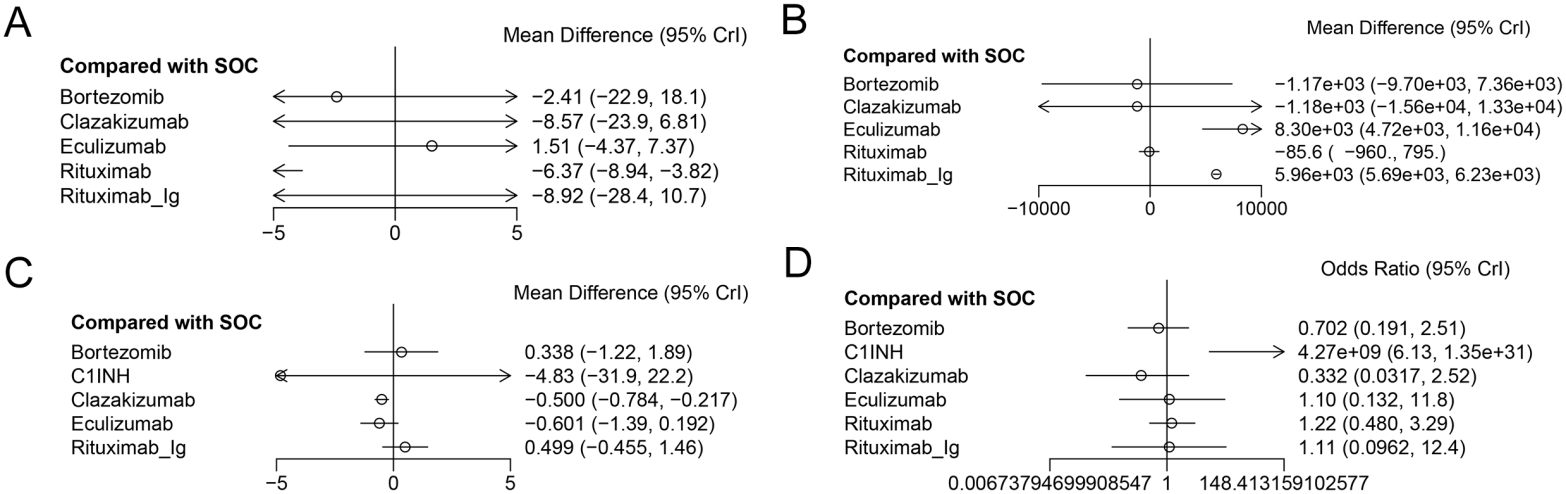
The forest plot of the studies accessing the efficacy of AMR in the context of drug therapy on **(A)** EGFR, **(B)** MFI, **(C)** G-score, **(D)** Infection.

**Figure 5:**
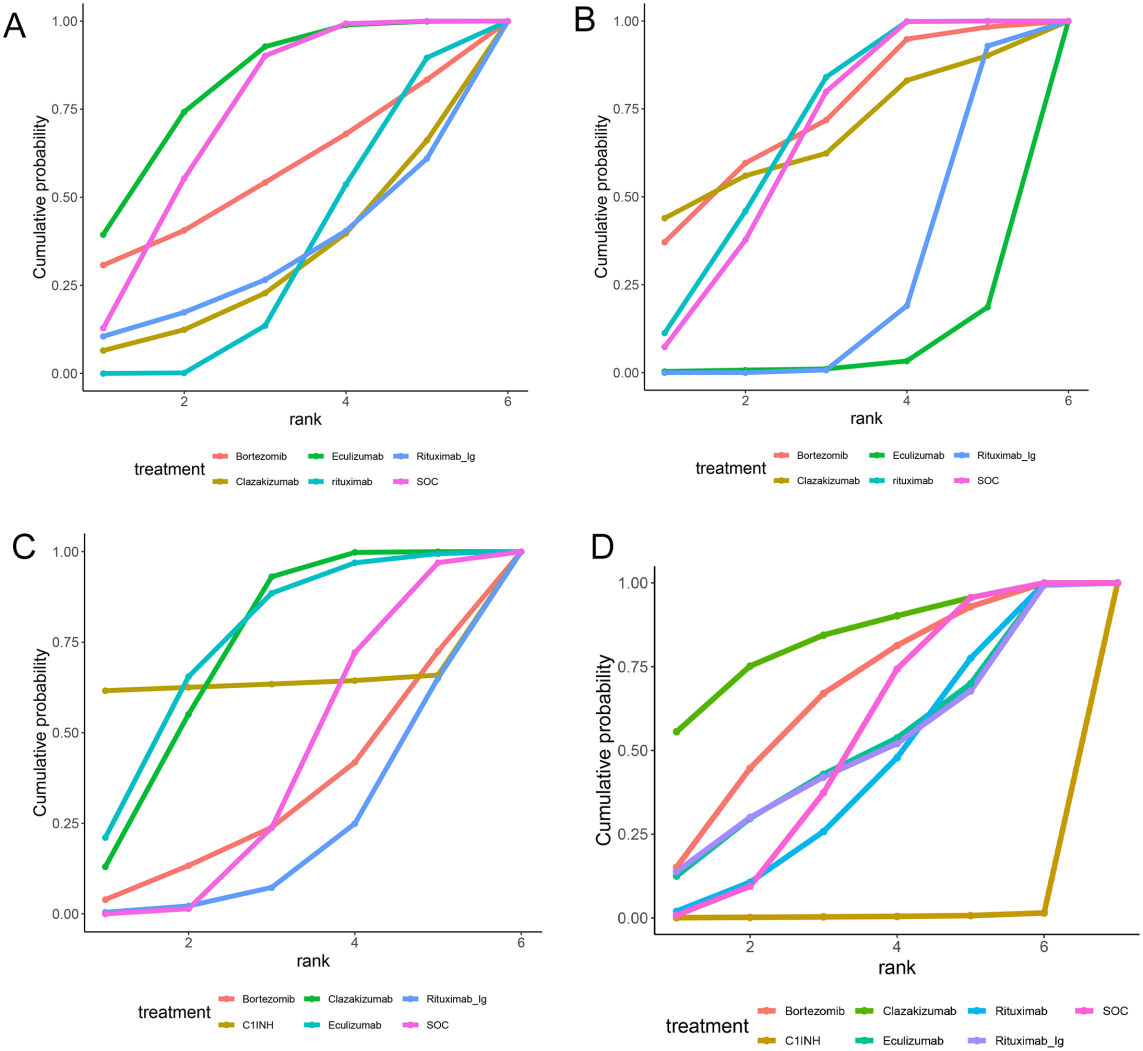
Area under the cumulative probability curve. **(A)** EGFR, **(B)** MFI, **(C)** G-score, **(D)** Infection.

A total of 5 RCTs reported end-point data for eGFR. The results of the NMA indicated that the increase in eGFR observed in the eculizumab group was greater than that observed in the control group. However, bortezomib, rituximab-Ig, and clazakizumab demonstrated a comparatively lower increase in eGFR in comparison to the control group. Further details are presented in **Figure 4A** and **Table 2**. The SUCRA scores for the treatments are as follows: eculizumab (81) > SOC (71.5) > bortezomib (55.4) > rituximab-Ig (31.2) > clazakizumab (29.5).Further details are presented in **Table 3**. These results indicate that eculizumab is the most promising treatment for increasing eGFR in transplant patients with AMR. The cumulative probability ranking is presented in **Figure 5A**.

**Table 2:** League table.

MD 95%CI(EGFR)							
Bortezomib		** **	** **	** **	** **	** **	
6.15 (-19.47, 31.94)		Clazakizumab	** **	** **	** **	** **	
-3.92 (-25.27, 17.48)		-10.07 (-26.54, 6.37)	Eculizumab	** **	** **	** **	
3.98 (-16.67, 24.62)		-2.21 (-17.73, 13.45)	7.88 (1.5, 14.26)	Rituximab	** **	** **	
6.51 (-21.94, 34.98)		0.35 (-24.52, 25.26)	10.43 (-10, 30.76)	2.54 (-17.33, 22.17)	Rituximab_Ig	** **	
-2.41 (-22.88, 18.12)		-8.57 (-23.92, 6.81)	1.51 (-4.37, 7.37)	-6.37 (-8.94, -3.82)	-8.92 (-28.41, 10.72)	SOC	
MD 95%CI(MFI)							
Bortezomib		** **	** **	** **	** **	** **	
61.21 (-16728.48, 16789.64)		Clazakizumab	** **	** **	** **	** **	
-9558.11 (-18833.57, 231.51)		-9609.07 (-24476.87, 5676.36)	Eculizumab	** **	** **	** **	
-1053.76 (-9608.24, 7512.73)		-1115.64 (-15548.95, 13383.49)	8570.81 (4092.09, 12054.19)	Rituximab	** **	** **	
-7094.81 (-15610.08, 1435.51)		-7155.47 (-21566.37, 7308.24)	2532.01 (-1812.6, 5915.16)	-6042.08 (-6960.17, -5127.19)	Rituximab_Ig	** **	
-1135.29 (-9655.54, 7384.43)		-1198.68 (-15602.13, 13267.11)	8488.91 (4150.94, 11855.75)	-85 (-962.21, 786.63)	5958.2 (5685.13, 6231.87)	SOC	
MD 95%CI(G-score)							
Bortezomib		** **	** **	** **	** **	** **	
5.17 (-21.91, 32.27)		C1INH	** **	** **	** **	** **	
0.84 (-0.74, 2.42)		-4.34 (-31.38, 22.71)	Clazakizumab	** **	** **	** **	
0.94 (-0.81, 2.68)		-4.23 (-31.28, 22.8)	0.1 (-0.74, 0.95)	Eculizumab	** **	** **	
-0.16 (-1.99, 1.66)		-5.33 (-32.38, 21.7)	-1 (-2, 0)	-1.1 (-2.35, 0.15)	Rituximab_Ig	** **	
0.34 (-1.22, 1.89)		-4.83 (-31.89, 22.21)	-0.5 (-0.78, -0.22)	-0.6 (-1.39, 0.19)	0.5 (-0.46, 1.46)	SOC	
OR 95%CI(Infection)							
Bortezomib	** **		** **	** **	** **	** **	** **
0 (0, 0.12)	C1INH		** **	** **	** **	** **	** **
2.12 (0.19, 30.32)	14272838194.64 (16.24, 2.76406640256832e+31)		Clazakizumab	** **	** **	** **	** **
0.64 (0.04, 7.61)	3991025975.07 (4.59, 7.78919590195908e+30)		0.3 (0.01, 5.94)	Eculizumab	** **	** **	** **
0.57 (0.11, 2.8)	3642297926.23 (4.95, 6.99516598857345e+30)		0.27 (0.02, 2.64)	0.89 (0.09, 11.26)	Rituximab	** **	** **
0.62 (0.04, 9.81)	4001960270.4 (4.28, 7.39680918837127e+30)		0.29 (0.01, 7.13)	0.98 (0.04, 28.17)	1.09 (0.08, 15.17)	Rituximab_Ig	** **
0.7 (0.19, 2.51)	4540076077.26 (6.25, 8.16675551621347e+30)		0.33 (0.03, 2.62)	1.09 (0.13, 11.64)	1.23 (0.48, 3.3)	1.13 (0.1, 13.03)	SOC

Both light gray and dark gray mean p<0.05.

**Table 3:** SUCRA ranking.

Treatment	eGFR	MFI	G-score	Infection
Bortezomib	55.4	72.3	31.1	66.8
Clazakizumab	29.5	67.1	72.2	83.5
Eculizumab	81.0	4.82	74.3	51.4
Rituximab	31.4	68.2	–	43.9
Rituximab_Ig	31.2	22.5	20.0	50.9
C1INH	–	–	63.6	0.55
SOC	71.5	65.0	38.9	52.9

#### 3.5.2 MFI

A total of 5 RCTs reported end-point data for MFI. The results of the NMA indicated that the decrease in MIF observed in the bortezomib, rituximab, and clazakizumab groups was greater than that observed in the control group. However, rituximab_Ig and eculizumab exhibited a comparatively lower reduction in MFI in comparison to the control group. Further details are presented in **Figure 4B** and **Table 2**. The SUCRA scores for the treatments are as follows: bortezomib (72.3) > rituximab (68.2) > clazakizumab (67.1) > SOC (65) > rituximab-Ig (22.5) > eculizumab (4.82). Further details are presented in **Table 3**. These results indicate that bortezomib is the most promising treatment for reducing MFI in transplant patients with AMR. The cumulative probability ranking is presented in **Figure 5B**.

#### 3.5.3 G-score

A total of 5 RCTs reported end-point data for the g-score. The results of the NMA indicated that the decrease in g-score observed in the eculizumab, clazakizumab, and C1INH groups was greater than that observed in the control group. However, bortezomib and rituximab-Ig exhibited a comparatively lower reduction in g-score in comparison to the control group. Further details are presented in **Figure 4C** and **Table 2**. The SUCRA scores for the treatments are as follows: eculizumab (74.3) > clazakizumab (72.2) > C1INH (63.6) > SOC (38.9) > bortezomib (31.1) > rituximab-Ig (20). Further details are presented in **Table 3**. These results indicate that eculizumab is the most promising treatment for reducing g-score in transplant patients with AMR. The cumulative probability ranking is presented in **Figure 5C**.

#### 3.5.4 Infection

All RCTs reported the occurrence of infection. The results of the NMA indicated that the clazakizumab and bortezomib groups are more effective than the control group in reducing infection rates. However, eculizumab, rituximab, and C1INH exhibited a higher incidence of infection in comparison to the control group. Further details are presented in **Figure 4D** and **Table 2**. The SUCRA scores for the treatments are as follows: clazakizumab (83.5) > bortezomib (66.8) > SOC (52.9) > eculizumab (51.4) > rituximab (43.9) > C1INH (0.55). Further details are presented in **Table 3**. These results indicate that clazakizumab is the most promising treatment for reducing infection in transplant patients with AMR. The cumulative probability ranking is presented in **Figure 5D**.

### 3.6 PB assessment

**Figure 6:**
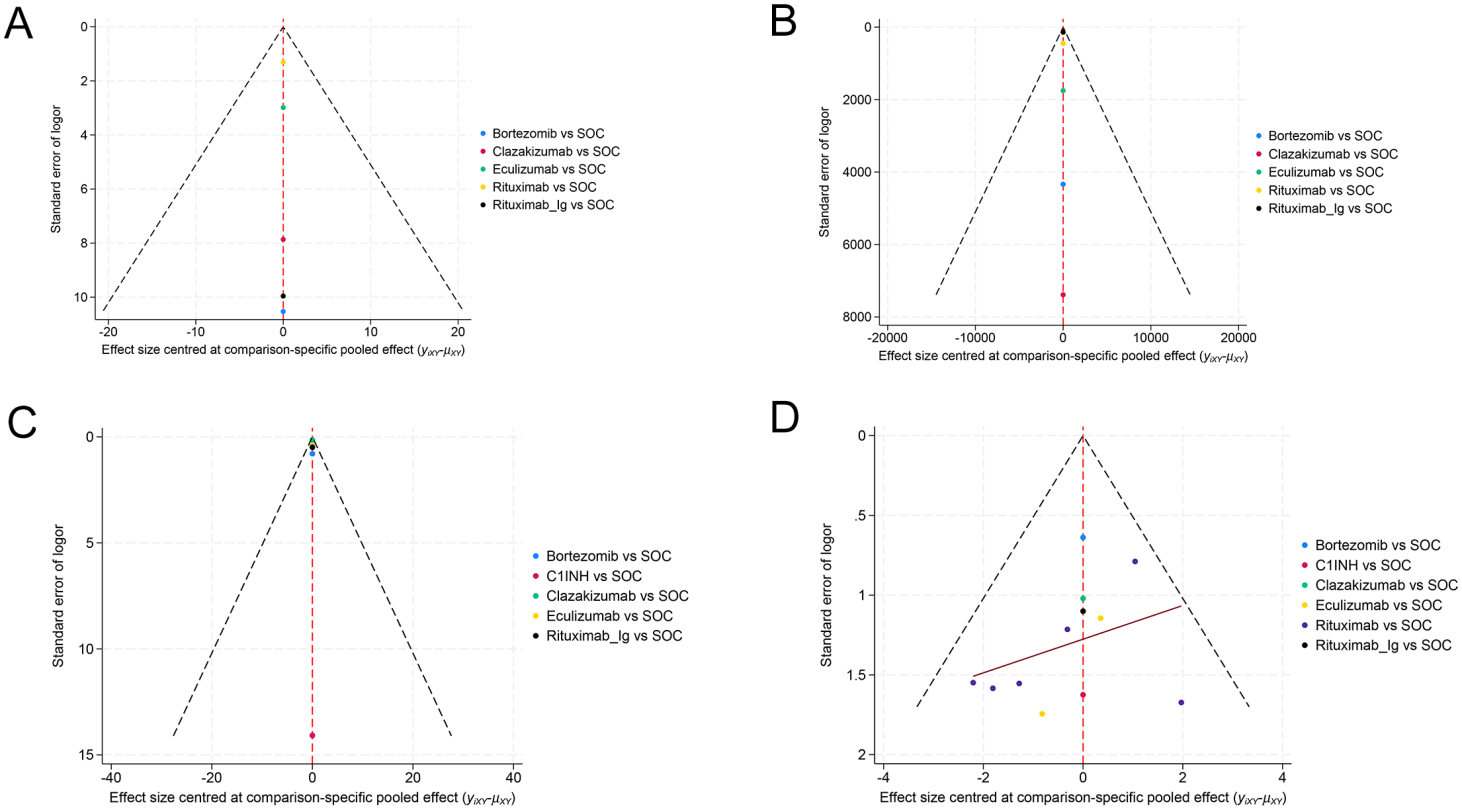
Funnel plot for evaluating publication bias. **(A)** EGFR, **(B)** MFI, **(C)** G-score, **(D)** Infection.

The PB funnel plot (**Figure 6**) indicates that the funnel plots of the two outcome indicators are not entirely symmetric. Some points fall outside the funnel plot, particularly at the bottom, which suggests the presence of a small sample effect and PB in this study. Consequently, it is imperative to interpret the findings of this study with a degree of caution.

## 4 Discussion

Currently, there is a great deal of clinical interest in the treatment of graft injury caused by AMR. Severe graft impairment has a profound impact on the function of the transplant organ and the survival of the transplant patient (19). Consequently, improving graft survival represents a crucial objective for transplant patients with AMR. There is currently no consensus on the most effective treatment for AMR after organ transplantation (5, 20, 21). This is due to the lack of RCTs, which is largely attributable to difficulties in patient recruitment (5). A significant number of studies fail to differentiate between various treatment modalities, thereby limiting their applicability to medical practice (22, 23). The objective of this study is to comprehensively examine the effectiveness of numerous pharmacological interventions in graft injury due to AMR.

During the literature screening process, it was observed that eGFR, MFI, g-score, and infection, among other factors, are commonly utilized for assessing the efficacy and safety of AMR in the context of drug therapy.

The NMA showed that eculizumab had a favorable impact on eGFR elevation. Bortezomib, rituximab, and clazakizumab demonstrated the potential to reduce MFI. Among these, bortezomib appears to be the most efficacious. Both eculizumab and clazakizumab have the potential to reduce the g-score. Among these, eculizumab appears to be the most efficacious. Both clazakizumab and bortezomib have the potential to reduce the incidence of infection in transplant patients with AMR. Among these, clazakizumab appears to be the most efficacious.

The role of complement has been recently highlighted in AMR after organ transplantation (24, 25). Eculizumab is a monoclonal antibody that is directed against C5. In a case report by Hassib Chehade (26) et al., eculizumab was administered early in the treatment of acute AMR in a highly sensitized kidney transplant recipient. This singular case study exemplifies the potential efficacy of early eculizumab administration in rapidly reversing severe AMR in pediatric transplantation, while maintaining excellent allograft function with undetectable circulating DSA levels. A novel therapeutic approach utilizing eculizumab in the management of acute AMR is proposed. In a study conducted by Ashley A. Vo (11) et al., it was found that eculizumab improved graft survival in patients, particularly in the event of severe AMR. Coralina Bernuy-Guevara (27) et al. found in an NMA that eculizumab prevented acute AMR in kidney transplant recipients. This will facilitate the more judicious use of complement inhibitors and enable the administration of this type of drug therapy on an individualized basis. In a randomized clinical trial conducted over a six-month period, S. Kulkarni et al. (28) demonstrated that eculizumab improves the eGFR in patients with AMR. Nevertheless, they underscored the need for further studies to ascertain which patients may benefit from this therapeutic intervention. In the review article, Melissa Y. Yeung (9) et al. observed that the eculizumab treatment group exhibited indications of enhanced eGFR compared to the control group. This may be attributed to the potential of eculizumab treatment to stabilize renal function in patients with chronic persistent DSA. However, she emphasized the necessity for larger-scale studies with longer follow-up periods to ascertain the efficacy of graft function preservation. Similarly, Patrick Yerly (29) et al. reported a case of a cardiac recipient with acute heart failure due to advanced acute AMR and eight neonatal donor-specific antibodies (DSA), in addition to thymoglobulin and IVIG. Following the administration of eculizumab, without plasma exchange, the DSA was completely cleared. The clinical, biological, and pathological features of this heart recipient are identical to those observed in early renal AMR. This may indicate a significant role for the complement system in the pathogenesis of acute transplant injury. Nevertheless, further investigation is necessary to confirm this hypothesis. In summary, eculizumab plays a beneficial role in protecting graft function. It is important to note that the studies by Ch. Legendre (24) et al. and Sujung Heo et al. (30) indicate that eculizumab has not yet demonstrated efficacy in preventing delayed graft function. However, it has been shown to be highly effective in the treatment and prevention of atypical hemolytic and uremic syndrome.

Clazakizumab, which blocks interleukin-6 (IL-6), has emerged as a promising therapeutic option for AMR (31). In a phase 2, single-center, open-label study, Stanley C. Jordan (32) et al. observed that patients receiving monthly subcutaneous treatment with clazakizumab for 12 months exhibited reduced DSA and graft inflammation. Moreover, no significant safety issues were observed. In a 20-patient open-label pilot study, Ashley A. Vo (33) et al. found that clazakizumab was safe and associated with a significant reduction in HLA antibodies and a high transplant rate in highly sensitized patients. In a separate study, Stanley C. Jordan (7) et al. proposed that IL-6 production in vascular endothelial cells following allogenic immune activation may represent another potential pathway for vasculitis. This is because endothelial IL-6 may stimulate immune cell responses that could be inhibited by anti-IL-6 therapy. It is of note that anti-IL-6 therapy demonstrated the capacity to induce Treg (25) and Breg (34) cells *in vivo*, suggesting the potential significance of clazakizumab in the prevention and treatment of DSA development and allograft rejection. Goce Spasovski (35) et al. concluded from the study that the new IL-6 blocking drug (clazakizumab) is a promising option for the prevention and treatment of AMR. It is inevitable that the clinical experience of tailoring immunosuppression to improve graft and patient survival for as long as possible will continue to be a significant factor in the field. Ashley Vo (36) et al. demonstrated that clazakizumab alone may enhance the efficacy and durability of desensitization therapy, as well as facilitate access to kidney transplantation in immunologically vulnerable patients. Similarly, Cynthia L. Miller (37) et al. demonstrated that IL-6/IL-6R signaling inhibition represents a novel therapeutic option for the prevention and treatment of allograft injury. In an RCT lasting 10 weeks, Konstantin Doberer et al. (38) demonstrated that clazakizumab is an effective and safe treatment for AMR. However, they also highlighted the importance of rigorous monitoring of the relevant patient indicators throughout the study. To date, clinical trials have demonstrated the efficacy of IL-6 blockade in the desensitization and treatment of AMR in kidney transplant recipients. The conclusions of Anita Borski (31) et al. are consistent with previous reports on the safety and efficacy of clazakizumab. In summary, it can be stated that clazakizumab is safe and has the potential to improve graft function in patients with AMR.

Bortezomib is a proteasomal inhibitor (PI), a novel AMR therapy that can delete plasma cells (19). In certain studies, bortezomib has also been demonstrated to be an efficacious primary AMR treatment. Jun Li (39) et al. demonstrated that rats treated with the broad-spectrum PI bortezomib exhibited a long-term reduction in allograft antibody production and histological improvement of the allograft. Plasma cells derived from allografted rats exhibit high levels of immunoproteasome expression. Bortezomib induces the accumulation of ubiquitin-binding, activation of the unfolded protein response, and induction of plasma cell apoptosis, thereby preventing the production of alloantibodies. Similarly, Hong Cheng (40) et al. established an animal model of AMR for kidney transplantation by conducting experiments in rats. Bortezomib has been demonstrated to reduce serum levels of DSA, alleviate peritubular capillary and glomerular inflammation post-transplantation, and to have a beneficial effect on C4d and IgG deposition, as well as on the number of B cells and plasma cells in peripheral blood and transplanted kidneys. The findings indicated that bortezomib increased the number of regulatory T cells and significantly reduced the proportion of helper T cells (Th17). In a study of refractory AMR, Janka Slatinska (13) et al. found that bortezomib treatment was well tolerated and effective in reducing HLA-B and HLA-DR antibody levels. However, it was not successful in depleting HLA-A and HLA-DQ DSA. The primary conclusion of Nicole S. Ejaz (41) et al. based on the PI bortezomib is that it represents a promising new protocol for the treatment of AMR, as it rapidly reduces immunodominant DSA and improves histology and renal function. The administration of bortezomib in a retreatment regimen has been demonstrated to provide a rapid, complete, and long-lasting elimination of DSA. Nils Lachmann (42) et al. demonstrated that graft survival rate, graft function, and DSA levels could be enhanced following bortezomib and high-dose IVIG treatment. Hiroto Egawa (43) et al. demonstrated that the PI bortezomib may be a promising treatment option due to its capacity to deplete plasma cell preparations. It is noteworthy that an RCT by Farsad Eskandary et al. (44) revealed that bortezomib did not significantly reduce DSA. Furthermore, they emphasized the need for large-scale, multicenter, controlled studies to further validate the efficacy and safety of bortezomib in relation to AMR. In conclusion, the combination therapy based on PIs provides a potential means for the rapid elimination of DSA in early acute AMR in renal transplant recipients.

Rituximab specifically binds to CD20 on the surface of B cells, resulting in the depletion of CD20-positive B lymphocytes through two pathways: antibody-dependent cell-mediated cytotoxicity (ADCC) and complement-dependent cytotoxicity (CDC). In a retrospective study, Hananeh Baradaran (45) et al. found that rituximab should be initiated as soon as possible in liver transplant recipients who experienced AMR, provided that no improvement in liver enzymes/bilirubin is observed during treatment strategies involving corticosteroids, plasma exchange, and IVIG. Sandesh Parajuli (46) et al. employed Kaplan-Meier analysis to demonstrate that the incorporation of rituximab was associated with superior graft survival. It was proved that the administration of rituximab in patients with advanced AMR could effectively reduce DSA and microcirculatory inflammation. Nora Schwotzer (47) et al. proposed that rituximab be employed as a B-cell immunomodulator to reduce DSA. The effective blocking of the terminal complement pathway may be a useful strategy for the treatment of acute AMR in allogeneic-sensitized recipients and may also be an effective strategy for xenotransplantation recipients. In a phase III, multicenter, double-blind, placebo-controlled trial conducted by Bénédicte Sautenet et al. (48), the administration of rituximab resulted in improvements in AMR, histological characteristics, and the Banff score at both the first and sixth months. While there were no statistically significant differences between the groups, the data indicated a potential advantage for the rituximab cohort. However, the authors noted that the study’s limited power may have led to the oversight of clinically meaningful differences between groups, indicating the need for additional trials with extended follow-up. Nevertheless, it is important to note that Puneet Sood (49) et al. and Elodie Bailly et al. (50) concluded that rituximab was ineffective in the treatment of AMR based on the results of a small sample size. Meanwhile, Francesc Moreso et al. (51) conducted a multicenter, prospective, randomized, placebo-controlled, double-blind clinical trial to evaluate the efficacy and safety of IVIG in combination with rituximab. The results indicated that the combination of IVIG and rituximab was ineffective in patients exhibiting renal transplant function and MFI.

C1 inhibitor (C1INH) plays a significant role in AMR after organ transplantation (52). Mel Berger (53) et al. has identified a growing body of evidence suggesting that complement is an important mediator of chronic AMR, which is the main cause of late graft loss. This evidence suggests that C1INH may also help protect the function of established grafts. Early clinical studies of transplantation have shown that C1INH has significant beneficial effects with minimal toxicity. Subsequently, Mel Berger (54) et al. suggested in his study report on kidney transplant recipients that C1INH treatment may reduce delay graft function. Other clinical studies and models indicate that C1INH may reduce sensitization and donor-specific antibody production and may improve the outcome of AMR, including in otherwise refractory patients. Ashley A. Vo (55) et al. found that C1INH was safe in the post-transplantation period, confirming that C1INH may prove useful in preventing AMR. Michael J. Eerhart (56) et al. found that recipients treated with C1INH showed less deposits of 3b/C5b-9 on day 7 biopsies. Animals treated with C1INH also tended to have prolonged mediated rejection-free survival. Vasishta S. Tatapudi (57) et al. proposed in two recent studies on the treatment of AMR that C1INH was well tolerated and associated with improved renal allograft function.

Meanwhile, a phase 2b multicenter, double-blind, randomized placebo-controlled pilot study was conducted by R. A. Montgomery et al. (58) to assess the efficacy of C1INH in treating AMR. The findings suggest that C1INH may play a beneficial role in the management of AMR. Based on these results, it is recommended that transplant recipients use C1INH complement blocking as a way to reduce graft damage and inflammatory response.

### 4.1 Limitations

First, the continuous variable data includes images, and there are some errors. Second, the number of included studies and the sample size was relatively small. Third, in order to confirm the conclusions of this study, it will be necessary to conduct further studies with larger sample sizes and multicenter designs.

## 5 Conclusions

The findings of this study indicate that eculizumab has the potential to enhance eGFR and reduce g-score. Bortezomib demonstrates greater efficacy in reducing MFI, while clazakizumab appears to be more effective in reducing infections.

Statements.

## Data Availability

The original contributions presented in the study are included in the article/**Supplementary Material**. Further inquiries can be directed to the corresponding author.
