# Supplementary material for: Network meta-analysis of pharmacological treatment for antibody-mediated rejection after organ transplantation
Source: Front Immunol. 2024 Dec 12;15:1451907. doi: 10.3389/fimmu.2024.1451907 (PMC11669588; doi:10.3389/fimmu.2024.1451907)
Supplement: Supplementary file 2 [file Table2.docx]

Supplementary Material 1. The specific search strategy.

PubMed search strategy

1. ((antibody-mediated rejection[Title/Abstract]) OR (antibody mediated rejection[Title/Abstract])) OR (humoral rejection[Title/Abstract])
2. randomized controlled trial[Publication Type] OR randomized[Title/Abstract] OR placebo[Title/Abstract]
3. (((antibody-mediated rejection[Title/Abstract]) OR (antibody mediated rejection[Title/Abstract])) OR (humoral rejection[Title/Abstract])) AND (randomized controlled trial[Publication Type] OR randomized[Title/Abstract] OR placebo[Title/Abstract])

Embases search strategy

1. 'antibody mediated rejection'/exp
2. 'antibody mediated rejection':ab,ti
3. 'antibody-mediated rejection':ab,ti
4. 'humoral rejection':ab,ti
5. 1 OR 2 OR 3 OR 4
6. 'random':ab,ti
7. 'placebo':ab,ti
8. 'double-blind':ab,ti
9. 6 OR 7 OR 8
10. 5 AND 9

WOB search strategy

1. TS=（antibody mediated rejection）OR TS=（antibody-mediated rejection）OR TS=（humoral rejection） and Preprint Citation Index (Exclude – Database)
2. TS=（random）OR TS=（placebo）OR TS=（double-blind）OR TS=（randomized）OR TS=（randomized controlled trial） and Preprint Citation Index (Exclude – Database)
3. 2 AND 1 and Preprint Citation Index (Exclude – Database)

Cochrane Library search strategy

1 (antibody mediated rejection):ti,ab,kw OR (antibody-mediated rejection):ti,ab,kw OR (humoral rejection):ti,ab,kw

Supplementary Material 2. The Deviance Information Criterion.

|  | consistency | non-consistency |
| --- | --- | --- |
| EGFR | 19.974261 | 19.971131 |
| MFI | 20.67182 | 20.79005 |
| G-score | 19.897334 | 19.924135 |
| Infection | 51.56659 | 51.65545 |
